# Commentary: L2 Phonology: Where Theory, Data, and Methods Meet

**DOI:** 10.3389/fpsyg.2021.774721

**Published:** 2021-12-13

**Authors:** Christine E. Shea

**Affiliations:** ^1^Department of Spanish and Portuguese, University of Iowa, Iowa City, IA, United States; ^2^Department of Linguistics, University of Iowa, Iowa City, IA, United States

**Keywords:** L2 phonology acquisition, L2 speech acquisition, phonology, L2 phonetics, phonetics and phonology

The field of L2 phonology studies the abstract representations created by L2 learners over the course of acquisition. L2 pronunciation, on the other hand, addresses concrete aspects of L2 speech, related to primarily to intelligibility, comprehensibility, and accentedness (Munro and Derwing, 1995). L2 phonology can be conceptualized as the frame in which L2 pronunciation develops or, where theory, data, and methods meet. In other words, pronunciation does not happen without phonology.

In their contribution “Developmental sequences in second language phonology: Effects of instruction on the acquisition of foreign sC onsets,” Cardoso, Collins and Cardoso examine how three different types of instruction interact with the production of /s/ + /l n t/ clusters by L1 Brazilian Portuguese/L2 English speakers (Cardoso et al., 2021). Previous research suggests that the order of acquisition of these non-native clusters will be affected by their internal sonority profile, or markedness, understood as relative degree of linguistic complexity [see Gass and Ard (1980) for early work on L2 syntax using universal hierarchies in the acquisition of relative clauses; (Cardoso and Liakin, 2009)]. From that perspective, /st/ clusters will be more difficult to acquire because there is minimal sonority fall from /s/ to /t/, compared to /s/ + /n/ or /l/ [see Yava (2010) for a similar analysis in L1 speech development]. Cardoso et al. ask whether the design of L2 pronunciation teaching should follow this natural, or universal order of acquisition and focus first on clusters that are predicted to be more easily acquired (/s/ + /n/ or /l/). Alternatively, research from L1 phonological development in disordered populations has shown that focusing first on more complex structures cascades down to the acquisition of less-marked structures (Gierut, 1999). A third possibility is also considered by Cardoso et al., where both complex and simple structures are taught together. Their results show that the pedagogical intervention emphasizing the most marked structure led to the greatest improvement in production of that cluster (st/) and also benefitted production of the other two clusters. Thus, the authors conclude that teaching oriented to more complex structures can lead to the acquisition and development of less marked structures as well. The results from Cardoso et al. show that abstract universal concepts such as sonority and phonotactic constraints influence L2 phonological acquisition and crucially, interact with explicit classroom learning. This has important implications for classroom pronunciation materials design, which typically does not take into account phonological universals of this type.

Speech-sound complexity is also at play in the contribution from Stefanich and Cabrelli (S&C) “The Effects of L1 English Constraints on the Acquisition of the L2 Spanish Alveopalatal Nasal” (Stefanich and Cabrelli, 2021). The authors examine the acquisition of the Spanish sound /ɲ/ by beginner and advanced L1 English speakers. The sound /ɲ/ occurs in syllable-onset position in Spanish words such as *montaña* “mountain” and *año* “year” and is not part of the English phonemic inventory. The closest English sound is the heterosyllabic sequence [n.j] in words such as *canyon*^1^. Their findings show that while the new contrast is learnable, neither group produced the target forms in the same way as the L1 Spanish speakers. The authors examined the production of F1 and F2 formant cues across the different groups. A rather unexpected finding was that the advanced group relied upon L1 representations to a greater extent than the beginner group. S&C speculate that advanced L2 learners' representations may, in fact, reflect a U-shaped development pattern; that is, the advanced learners have realized that /ɲ/ is not /nj/, but they are still not able to produce [ɲ] in target-like fashion and revert to the closest L1 sound (Tessier, 2019).

Zhang and Levis in their article “The Southwestern Mandarin /n/-/l/ merger: Effects on production in Standard Mandarin and English” examine how speakers of Southwestern Mandarin, a dialect of Mandarin in which the contrast between [l] and [n] is merged, produce these segments in initial and word-medial positions in L3 English and L2 Standard Mandarin. The results from a reading task revealed an interesting asymmetry with respect to the /n/-/l/ merger whereby in English, participants neutralized in favor of [l] while in Standard Mandarin, neutralization was in favor of [n], suggesting an interaction between the order of language acquisition (L2 vs. L1) and potentially, the role of each sound in the phonological system of each language.

These three studies make interesting and important contributions to the field of L2 phonological development in terms of how abstract structure affects the order of acquisition (Cardoso et al., 2021), syllable structure (S&C), and L2 vs. L3 neutralization asymmetries (Zhang and Levis, 2021). Moving forward, I would encourage researchers in the field of L2 phonology to consider two important additional factors related to (a) the type of data collected and (b) the pool of participants.

In terms of the type of data collected, the field of L2 phonology would benefit greatly from more studies focused on longitudinal development. While recent studies have examined longitudinal phonetic development (Nagle, 2019; Casillas, 2020), there are relatively few that use empirical, quantitative data to analyze phonological development in this way (e.g., changes over time in syllable structure representation, such as that examined by Cardoso et al. and S&C). As well, researchers should consider the longitudinal development of perception and production together [Nagle, 2021; see Nagle and Baese-Berk (2021) for an overview] to determine how tightly coupled these may be at the phonological level.^1^

Related to this is a call for a clearer definition of what is meant by “phonological representations.” S&C provide a good example of how future researchers may go about this in their contribution. These authors establish clear hypotheses regarding changes in the representation of the alveopalatal nasal and how, as the learner gains experience with the target language, each change in representation might manifest in production. Furthermore, S&C also characterize what learners needed to adjust in terms of their syllable representations to successfully produce the alveopalatal nasal. Not every study that purports to examine L2 phonology is as clear on the learning task, possible outcomes and implications for representational claims.

Both Cardoso et al. and S&C address issues related to complexity and re-alignment of L1 phonological representations as part of the task facing their participants. I would encourage future researchers to consider these issues as they relate to Heritage Speakers (HS). While HS are (of course) distinct from L2 learners, who are the focus of this Research Topic, data from this group of speakers can contribute meaningfully to the development of phonological representations because (a) HS are naturalistic learners and (b) HS undergo a shift in language dominance at some point during childhood, due to a decline in the amount of input received. The study of HS phonological development in both the heritage and dominant language would allow researchers to consider age and input as separate factors (Flege, 2018; Flege and Bohn, 2021; p.c. B. McMurray) and help understand the way phonological development is affected by each.

Finally, future research should also consider how phonology relates to the real-time unfolding of speech cues in lexical recognition. Crucially, L2 (and L3) phonological representations are only “functional” in so far as they encode lexical items, or, at the very least, can serve as potential representations for the target language (Darcy et al., 2013; Cook et al., 2016). Recent work has shown that both phonology and phonetics play a key role in lexical encoding of contrasts. Combining this with studies examining how cues to, say, syllable structure unfold in real time and affect lexical competition in bilinguals (Sarrett et al., 2021) is necessary to gain a fuller picture of L2 phonological development.

## Author Contributions

The author confirms being the sole contributor of this work and has approved it for publication.

## Conflict of Interest

The author declares that the research was conducted in the absence of any commercial or financial relationships that could be construed as a potential conflict of interest.

## Publisher's Note

All claims expressed in this article are solely those of the authors and do not necessarily represent those of their affiliated organizations, or those of the publisher, the editors and the reviewers. Any product that may be evaluated in this article, or claim that may be made by its manufacturer, is not guaranteed or endorsed by the publisher.

## Footnotes

^1^Phonotactic constraints in English prohibit homosyllabic ^*^[.nj] sequences in most dialects (Kulikov, 2010). Words such as *news* [njuz] can be produced with the [nj] sequence in palatizing dialects.
